# Stigma Functionality and Fertility Are Reduced by Heat and Drought Co-stress in Wheat

**DOI:** 10.3389/fpls.2019.00244

**Published:** 2019-03-07

**Authors:** Attila Fábián, Eszter Sáfrán, Gabriella Szabó-Eitel, Beáta Barnabás, Katalin Jäger

**Affiliations:** Plant Cell Biology Department, Agricultural Institute, Centre for Agricultural Research, Hungarian Academy of Sciences, Martonvásár, Hungary

**Keywords:** anatomy, fertility, heat and drought co-stress, morphology, RNS, ROS, stigma, wheat

## Abstract

As a consequence of climate change, unpredictable extremely hot and dry periods are becoming more frequent during the early stages of reproductive development in wheat (*Triticum aestivum* L.). Pollen sterility has long been known as a major determinant of fertility loss under high temperature and water scarcity, but it will be demonstrated here that this is not the exclusive cause and that damage to female reproductive organs also contributes to losses of fertility and production. Changes in the phenology, morphology, and anatomy of female reproductive cells and organs, in the ROS and RNS generation of stigmatic papilla cells, and in fertility and yield components in response to simultaneous high temperature and drought at gametogenesis were studied in two wheat genotypes with contrasting stress responses. The combination of high temperature (32/24°C) and total water withdrawal for 5 days at gametogenesis altered the phenology of the plants, reduced pollen viability, modified the morphology and the anatomy of the pistils, enhanced the generation of ROS and RNS, intensified lipid peroxidation and decreased the NO production of stigmatic papilla cells, all leading to reduced fertility and to production loss in the sensitive genotype, depending on the position of the floret on the spike. Reduced functionality of female and male reproductive parts accounted for 34% and 66%, respectively, of the total generative cell- and organ-triggered fertility loss.

## Introduction

Wheat has a leading role in human nutrition and animal feed in the world with the largest harvested area (220.1 million hectares) and the second largest production (749.5 million tons) among cereals (FAOSTAT, 2016). With the continuously rising human population of the planet and the constant decline in agricultural land availability and quality, forecasted trends of yield increase will not be sufficient to satisfy future demand (Ray et al., 2013; Zandalinas et al., 2018). The enhancement of yield stability even under unfavorable environmental conditions is one of the primary goals of wheat breeders (Lamaoui et al., 2018). Among the extreme weather events, high temperature and drought are expected to be the main yield decreasing factors (IPCC, 2014; Lesk et al., 2016). Globally, drought accounts for 21% yield loss on average (Daryanto et al., 2016). A 1°C increase in global temperature could reduce the global wheat yield by 4.1–6.4% depending on the method used for yield projection (Liu et al., 2016). It has been reported that more than 40% yield fluctuation of wheat can be attributed to climate change (heat waves and drought) at the global, national, and subnational scales (Zampieri et al., 2017). The growth, physiological, and metabolic responses of plants to a combination of heat and drought (HD) stresses are unique and cannot be directly extrapolated from the responses to each of these stresses separately (Rizhsky et al., 2002, 2004). Different stress combinations should be handled as a new state of stress in plants, requiring novel types of defense and acclimation responses (Suzuki et al., 2014).

The sensitivity of a plant to environmental factors depends on the species, genotype and developmental stage, and on the duration and severity of the stress. Heat and drought stress during reproductive development may seriously affect crop yields (Barnabás et al., 2008), which can be attributed especially to the high sensitivity to stress shown by pollen development (Saini and Aspinall, 1981; Saini et al., 1984; Lalonde et al., 1997; Jäger et al., 2008; for reviews, see Dolferus et al., 2011; De Storme and Geelen, 2014). Compared to pollen dysfunction, the significance of the damage sustained by the physically better protected female reproductive cells and organs is generally considered as a minor factor in yield loss (Hedhly, 2011; Giorno et al., 2013); therefore less attention has been paid to the heat and drought sensitivity of their development.

Although there are emerging evidences of the sensitivity of female reproductive cell and organ development to heat or drought stress *per se* in sorghum, rice, maize, wheat, tomato, and canola (Saini and Aspinall, 1981; Saini et al., 1983; Mitchell and Petolino, 1988; Polowick and Sawhney, 1988; Jagadish et al., 2010; Prasad et al., 2011; Onyemaobi et al., 2017; Djanaguiraman et al., 2018; Pan et al., 2018), no information is available on the combined effect of these two stresses. Majority of studies focus on the effect of heat or drought stress during meiosis and anthesis and little attention has been paid to gametogenesis. During this process, if undisturbed, the sexual organs and gametes complete their development, reach their final size and accumulate the starch reserves needed for successful fertilization and the nourishment of the first cell division cycles of the embryo and the endosperm.

Despite their central role in plant reproduction, the vulnerability of wheat pistils to heat or drought stress has hardly been investigated to date (Saini and Aspinall, 1981, 1982; Saini et al., 1983; Prasad and Djanaguiraman, 2014; Onyemaobi et al., 2017). Saini and Aspinall (1982) and Saini et al. (1983) reported reduced fertility and altered ovary and ovule development in 30% of wheat pistils as a consequence of high temperatures during meiosis. Wheat plants, similarly to other Gramineae species, possess two-branched, feathery, dry plumose type stigmas (Heslop-Harrison and Heslop-Harrison, 1980; Heslop-Harrison, 1992). The stigma tissues have multiple tasks during pollination, all of which are crucial for successful fertilization: the capture and hydration of the pollen, pollen tube guidance and transmission (Heslop-Harrison, 1979). The first three of these four cardinal steps occur on the receptive secondary branches of the stigma. In wheat, these branches are composed of four rows of highly vacuolated papilla cells, with a centrally located nucleus and a thin layer of marginal cytoplasm (Heslop-Harrison and Heslop-Harrison, 1980). Although Prasad and Djanaguiraman (2014) found that wheat stigmas and ovaries became desiccated following exposure to high temperature for 5 days before anthesis and that the pollen capturing ability of the stigma decreased, no information was given on the structural changes and processes underlying this phenomenon. However, the stigma, which plays an essential role in reproductive processes, is the most delicate but the least protected female organ, making it the most sensitive to adverse environmental conditions. If receptive, it provides the exact conditions required for pollen germination and the sustained growth and guidance of the pollen tube through the pistil and ovary (Heslop-Harrison, 2000), but no information is available on the effect of HD co-stress on its anatomy and functionality.

Both extreme high temperatures and water shortage lead to the excessive generation of reactive oxygen species (ROS) and reactive nitrogen species (RNS), which function as signal transduction molecules, but can also cause extensive cellular damage when the balance between the production and scavenging of these compounds is impaired (Hasanuzzaman et al., 2012; Choudhury et al., 2017; Zandalinas et al., 2018). ROS and RNS are partially reduced or activated forms of molecular oxygen and nitrogen (del Río, 2015). Small amounts of these radicals and compounds are produced continuously even under favorable conditions, particularly in the plastids, mitochondria, peroxisomes, cytosol, and apoplast. The most important types of reactive radicals and compounds are singlet oxygen (^1^O_2_), superoxide anion (O_2_^•-^), hydrogen peroxide (H_2_O_2_), hydroxyl radical (OH^•^), nitric oxide (NO), and peroxynitrite (ONOO^•-^; Molassiotis and Fotopoulos, 2011; Demidchik, 2015). These molecules differ greatly in their lifespan, on a nanoseconds to seconds scale. ROS and RNS also show diverse reactivity, from moderate (O_2_^•-^) to very high (OH^•^, ONOO^•-^; Waszczak et al., 2018), being able to oxidize lipids, proteins, carbohydrates and nucleic acids, therefore effectively impairing the structural integrity of cells when present in large amounts (Vandelle and Delledonne, 2011; Demidchik, 2015). On the other hand, the signaling role of ROS and RNS has been revealed in both developmental and stress reaction processes in the past decade (Waszczak et al., 2018). Although a certain amount of information is available on the role of the ROS content of the stigma and stigmatic papillae in developmental changes and pollen incompatibility processes (McInnis et al., 2006; Serrano et al., 2010, 2012, 2015; Domingos et al., 2015; Zafra et al., 2016), there are no data on the environmental stress-induced ROS and RNS generation in this delicate and important organ. As generative processes show significant vulnerability to heat and drought stress, it can be hypothesized that ROS and RNS play an important role in the reduction in fertility and in consequent yield loss.

The sensitivity of female reproductive tissues to simultaneous heat and drought stress is not well understood. Addressing the morphological, anatomical, physiological, and molecular mechanisms conferring sensitivity and tolerance to HD co-stress will help to develop wheat genotypes capable of adapting to a changing climate. Hence, the objectives of this study were to (1) reveal the combined effect of heat and drought co-stress during gametogenesis on the morphology, structure and functionality of female reproductive cells and organs, and on the yield components of wheat genotypes with contrasting HD tolerance; (2) shed light on the HD stress induced ROS and RNS generation of stigmatic papilla cells, and (3) unravel the link between HD stress-induced oxidative damage and fertility loss.

## Materials and Methods

### Plant Material, Plant Cultivation, and Stress Conditions

**FIGURE 1:**
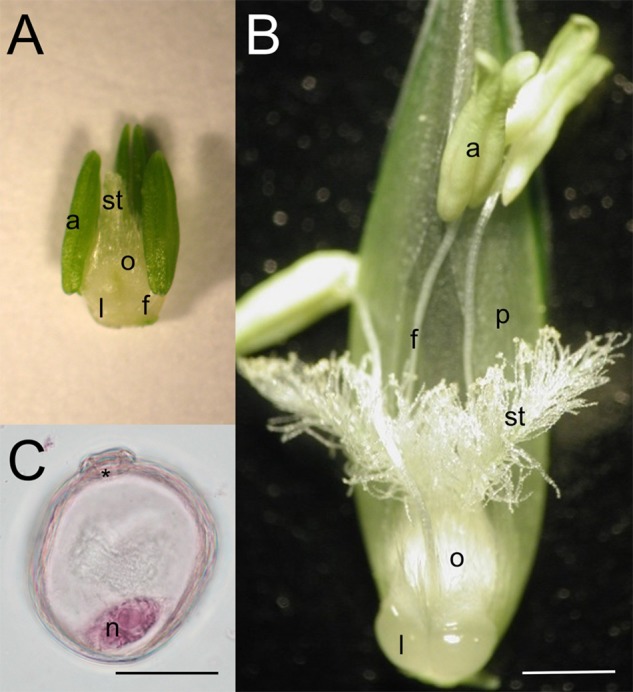
Morphology of wheat reproductive organs and male reproductive cells of the Cappelle Desprez winter wheat variety at **(A,C)** the mid-uninucleate stage of microspore development and **(B)** anthesis. a, anther; f, filament; l, lodicule; n, nucleus; o, ovary; p, palea; st, stigma; asterisk, aperture. Bar represents **(A,B)** 1 mm; **(C)** 25 μm.

Two winter wheat genotypes, the drought-tolerant Plainsman V^1^ and the drought-sensitive Cappelle Desprez^2^ were used in the experiments. In previous studies (Jäger et al., 2008, 2014; Fábián et al., 2011) significant differences were found in the fertility loss induced by heat and drought stress in the varieties Cappelle Desprez and Plainsman V, making them suitable for examining the effect of HD co-stress on female reproductive cells and organs. Plants (*n* = 50 per genotype and treatment) were planted in pots containing 2 kg of a soil-sand-peat mixture (3:1:1, v/v/v) after 7 weeks of vernalization at a temperature of 4°C, and grown in growth chambers (Conviron, Winnipeg, Canada) using the spring climatic program T1 (Tischner et al., 1997) under optimum environmental conditions until the mid-uninucleate stage of microspore development (hereinafter referred to as MU). The max/min day/night temperature in the growth chambers was 22/14°C, and irrigation was carried out regularly in the morning at a rate of 150 ml/day. The pots were randomly arranged and then rearranged every week to reduce border effects and minimize any variation in light and temperature. The fact that the microspores were in the MU stage was checked by acetocarmine staining followed by light microscopy (Figure 1) and based on the distance between the auricles of the flag leaf and the penultimate leaf. Plants with main spikes at MU were identified each morning, tagged and transferred to control or stress chambers. HD stress was generated by total water withholding at 32/24°C max/min temperature for 5 days from MU until flowering under controlled conditions. During this period the mean volumetric water content of the soil dropped from 37.2 to 5.1% that was equal to 13 kPa and 10,549 kPa soil water potential, respectively. The relative daily max/min air humidity was 75%/65% and 65%/30% in the control and stress chambers, respectively. The light intensity during the experiment was 300 μmol m^-2^ s^-1^. At 10 am on the day of flowering (at the end of the 5-day treatment) samples were collected and 20 plants of each genotype and treatment were re-irrigated and grown to full maturity under control conditions at a final max/min temperature of 32/24°C. If not otherwise indicated, all the below-mentioned measurements and samplings were carried out in at least three biological and three technical repetitions (per genotype and treatment) at anthesis. The effect of treatments was assessed for the tagged main tillers only.

### Measurement of Water Content

The volumetric water content of the soil (*n* = 10 pots per genotype and treatment) was monitored using an HH2 moisture meter connected to an SM200 soil water sensor (Delta-T Devices Ltd., Cambridge, United Kingdom) on the basis of the changes recorded in the apparent dielectric constant at full water saturation and during the treatments. The relative water content (RWC) of flag leaves excised from the main tillers of 15 plants per genotype and treatment was determined at the end of HD stress using the fresh weight (FW) at excision, the saturated weight (SW) after 24 h re-hydration in distilled water at 25°C in the dark, and the dry weight (DW) after oven drying for 24 h at 80°C. The leaf RWC was calculated using the following equation: $RWC(\%)=\frac{FW-DW}{SW-DW}\times 100$.

### Phenology

The days from MU to anthesis and physiological maturity were noted for each genotype. MU was determined as described above. A plant reached anthesis when the first anther appeared on the middle of the isolated and tagged main spike. Physiological maturity was reached when the peduncle of the main spike became yellow.

### Determination of Yield Components

The main tillers of control and drought-stressed plants (*n* = 20 per genotype and treatment) were hand-harvested and the spikes were threshed at full maturity. The plant height and the spikelet number, grain number and grain weight of both the basal and upper spike halves were determined and mean values were calculated for each treatment and cultivar. The thousand-grain weight (TGW) was calculated from the data. The fertility index was calculated as the quotient between the potential and actual grain number per spikelet. Three grains per a spikelet characteristic of both genotypes were considered when potential grain number of intact spikelets was determined. In case of truncated or manipulated spikes, two grains per spikelet were considered when potential grain number was calculated.

### Pollination Experiment

In order to shed light on the contribution of HD stress-induced stigma injury to fertility reduction in Cappelle Desprez, 90 plants were grown until MU under the control conditions described above. At MU the plants were divided into two groups. While the plants in the 1st control group were grown further under optimum conditions, plants of the second group were subjected to HD co-stress until flowering. Only the main tillers of mother plants were used in this experiment. The control group consisted of four sub-groups: (i) pollen donor plants (*n* = 30), half of which were planted 2 weeks earlier in order to compensate for the more rapid development of HD-stressed plants, (ii) plants with intact florets for free pollination (*n* = 15), (iii) pollen recipient plants in which the top third of the glumes, paleas and lemmas in all the primary and secondary florets were cut off and the central florets were removed 3 days before anthesis (truncated florets; DBA; *n* = 15) in order to simulate the majority of the damage sustained by the florets during emasculation, and (iv) pollen recipient plants treated as in (iii) and also emasculated 3 DBA (manipulated florets; *n* = 30). The treated group consisted only of groups ii, iii, and iv. Care was taken not to cut or touch the stigmas during these manipulations. All the spikes were bagged in order to prevent pollination by neighboring ones and the date of floret manipulation was recorded on crossing tags. At the onset of anthesis (dehiscent anthers visible in similarly developed spikes in group ii) both the control and HD-stressed main spikes with mutilated and emasculated florets (group iv) were hand-pollinated with pollen from the pollen donor control plants (group i) and were grown to maturity under optimum conditions. The fertility index of both sub-regions (base, top) of the main spikes was calculated and analyzed.

### Pollen Viability Assay

Wheat pollen collected separately from the base and top of the spikes was incubated in 0.1 M pH 7.2 Sorensen’s phosphate buffer containing 0.5% TTC for 15 min in the dark at 37°C. In this assay, the TTC is converted into red formazan dye by dehydrogenases if the pollen cells are viable. Pollen viability was calculated as the percentage of dark red pollen to the total number of counted pollen grains across 20 microscopic field views, such that at least 2,800 pollen grains were assessed per genotype and treatment.

### Morphometric Analysis of the Pistils

The pistils (*n* = 8 in Plainsman V; *n* = 10 in Cappelle Desprez) in one primary floret in each spikelet, located along one side of the rachis and numbered starting at the bottom, were collected from three plants per genotype and treatment at anthesis. The rudimentary florets located both at the base and top of the spikes were not taken into account. Micrographs were taken using a DiscoveryV8 stereomicroscope (Zeiss, Darmstadt, Germany) equipped with an HD Ultra camera (Euromex, Arnhem, Netherlands). The area of the ovaries and the length of the stigmas was measured using Image-Pro Plus 7.0 software (Media Cybernetics, Rockville, MD, United States).

### Light Microscope Studies: Pistil Anatomy

Stigmas (*n* = 4 per genotype and treatment) from the central regions of both the lower and upper halves of the spikes were excised prior to anthesis, incubated in Tris-HCl buffer (10 mM, pH 7.4) containing 5 μM Syto-63 fluorescent nucleic acid probe (Thermo Fisher Scientific) in the dark for 10 min at room temperature and washed for 3 min in Tris-HCl buffer. As Syto-63 stains the nucleus and the cytoplasm with different intensities it was used as a general stain for the visualization of the stigma papilla cell structure. Fluorescence was detected using a Leica SP8 confocal laser scanning microscope (Leica Microsystems GmbH, Wetzlar, Germany). Syto-63 was excited at 633 nm and the emitted fluorescence was detected at 650–700 nm. In addition, differential interference contrast (DIC) images of the papilla cells were collected during confocal image acquisition using a transmitted light detector.

For histological studies, pistils (*n* = 4 per floret position, genotype and treatment) isolated from central regions of both the lower and upper halves of the spikes were collected just before anthesis, fixed in 50 mM Na-cacodylate buffer (pH 7.2) containing 2.5% glutaraldehyde (v/v) and 4% formaldehyde (w/v) overnight at 4°C, washed, dehydrated in an ethanol series and gradually infiltrated with LR white acrylic resin (Ted Pella, Redding, CA, United States) according to the manufacturer’s instructions. The resin was polymerized under UV light at -20°C. Semi-thin sections (1 μm) were serially sectioned at the sagittal plane of the ovaries and at the transverse plane of the stylodia using an Ultracut-E microtome (Reichert-Jung, Heidelberg, Germany) and were stained with periodic acid-Schiff (PAS) and 1% Amido Black for polysaccharides and proteins, respectively. Stained sections were mounted in 50% glycerol containing 7% acetic acid, examined under a BX51 light microscope (Olympus, Tokyo Japan) and analyzed using an Image-Pro Plus 5.1 image analysis software (Media Cybernetics, Inc., Bethesda, MD, United States).

### Detection of Reactive Oxygen Species, Reactive Nitrogen Species, and Lipid Peroxidation in Stigmatic Papilla Cells

The ROS and RNS contents and the quantity of lipid peroxidation products were determined in the stigmas (*n* = 5 per genotype and treatment) isolated from the central regions of both the lower and upper halves of six main spikes. Samples were collected just prior to anthesis. Pistils with intact stigmas were incubated in Tris-HCl buffer (10 mM, pH 7.4; except labeling with C11-BODIPY where 60 mM Sorensen’s phosphate buffer pH 7.4 was used) containing the relevant fluorescent probe in the dark, followed by washing three times for 3 min in Tris-HCl buffer. The stigmas were carefully excised from the ovaries and the fluorescent signal was visualized immediately using a Leica SP8 laser scanning confocal microscope (Leica Microsystems GmbH, Wetzlar, Germany). General cellular oxidative stress was assessed using 2′,7′-dichlorodihydrofluorescein diacetate (H_2_DCFDA, Sigma) at 10 μM final concentration at 37°C for 30 min, with excitation at 488 nm and signal detection at 517–527 nm. Mitochondrial O_2_^•-^ was detected using MitoSOX Red dye (Thermo Fisher Scientific) at 5 μM final concentration at room temperature (RT) for 30 min, with excitation at 514 nm and signal detection at 580–700 nm. The superoxide anion radical (O_2_^•-^) content in the cytosol was labeled with dihydroethidium (DHE, Sigma) at 10 μM final concentration at 37°C for 30 min, with excitation at 514 nm and signal detection at 580 to 700 nm. Hydroxyl radical (OH^•^) and peroxynitrite (ONOO^-^) were monitored using aminophenyl fluorescein (APF, Sigma) at 10 μM final concentration at RT for 30 min, with excitation at 488 nm and signal detection at 500–550 nm. Extracellular hydrogen peroxide (H_2_O_2_) was detected using Ampliflu Red: samples were incubated with Ampliflu Red and horseradish peroxidase at 50 μM and 0.2 U/ml final concentration, respectively, at RT for 30 min, with excitation at 561 nm and signal detection at 565–650 nm. Intracellular H_2_O_2_ was visualized with dihydrorhodamine 123 (DHR 123, Thermo Fisher Scientific) at 5 μM final concentration at RT for 30 min, with excitation at 514 nm and signal detection at 520–600 nm. Nitric oxide (NO) was labeled with 4-amino-5-methylamino-2′,7′-difluorofluorescein diacetate (DAF FM-DA, Thermo Fisher Scientific) at 10 μM final concentration at RT for 60 min, with excitation at 488 nm and signal detection at 500–580 nm. Lipid peroxidation was monitored using C11-BODIPY^TM^ 581/591 (Thermo Fisher Scientific) at 1 μM final concentration at 37°C for 30 min. The fluorescent signals of the non-oxidized and oxidized forms were acquired using simultaneous excitation. The non-oxidized form of the dye was excited at 561 nm and signals were detected at 570–620 nm. The oxidized form of the probe was excited at 488 nm, signals were detected at 500 to 560 nm. The dimer formation of C11-BODIPY^TM^ molecules with red-shifted fluorescence (excimers; Pap et al., 1999) was monitored by fluorescence measurement at 570–630 nm after excitation at 488 nm. ROS- and RNS-sensitive fluorescent dyes were used simultaneously with ROS scavengers as negative controls and ROS generating treatments as positive controls for the proper evaluation of the fluorescence intensities detected in control and HD treated. Specific localization patterns of the used probes were evaluated during the preliminary experiments (Supplementary Table 1) according to the literature (Ortega-Villasante et al., 2016). All microscope settings (i.e., laser power, detector gain, detection spectra, etc.) were saved for each fluorescent probe and kept the same throughout all the repetitions.

### Quantification of Fluorescent Signals

Fluorescent signal intensities were measured on the images using Leica Advanced Fluorescence software v3.1.5.1638 (Leica Microsystems GmbH, Wetzlar, Germany). Relative fluorescence intensities were measured on 60 micrographs per genotype, treatment and fluorescent probe, using 10 regions of interest (ROIs) per micrograph containing only the organelles emitting specific signals (e.g., mitochondrion, cell wall, vacuole). Unspecific autofluorescence originating from cell walls was not taken into account during quantification. The measured data were normalized against the background fluorescence. In the case of Ampliflu Red, measured fluorescence intensity values were corrected by a factor of cell wall autofluorescence determined during our preliminary experiments on unstained control and HD treated papilla cells. The ratio of oxidized BODIPY^TM^ 581/591 C11 probes was calculated from pixel intensities measured in ROIs using the following equation (Pap et al., 2000):

oxidized% =Intensityoxidized(500−560nm)Intensityoxidized(500−560nm)+Intensitynon−oxidized(570−620nm)*100

### Statistical Analysis

All measurements were carried out in at least three biological and three technical repetitions. Data were subjected to ANOVA (SPSS version 16.0, IBM Corp., Armonk, NY, United States). The mean values were compared by the Tukey’s multiple range test taking *P* ≤ 0.05 as significant to compare the differences between treatments and genotypes. Pearson’s correlation coefficient was used to identify relationships between the measured characters. Mean values along with standard deviations are presented in the tables and figures.

## Results

### Reduced RWC of Wheat Plants After HD Stress

Combined heat and drought stress applied for 5 days prior to anthesis induced a substantial reduction in the RWC of the flag leaves in both genotypes, but this reduction in Cappelle Desprez RWC (43%) was significantly (*P* ≤ 0.05) more pronounced than in Plainsman V (21%; Table 1). The leaves of treated Cappelle Desprez plants showed increasing visible symptoms of dehydration, starting around noon on the third day of treatment, while leaf rolling was only observed on the flag leaves of treated Plainsman V plants on the fifth day of treatment.

**Table 1:** Relative water content of control and HD-stressed Cappelle Desprez and Plainsman V flag leaves at anthesis.

Genotype	Flag leaf water content (%)	
	
	Control	HD stress
Plainsman V	88.61 ± 4.27^a^	70.03 ± 4.94^b^
Cappelle Desprez	87.58 ± 1.13^a^	50.12 ± 1.38^c^


*Values represent means ± standard deviations. Means with different superscripts are significantly different at least at the *P* ≤ 0.05 level of probability. HD, simultaneous heat and drought.*

### Simultaneous Heat and Drought Reduced Fertility and Production

The main spikes of the Cappelle Desprez variety were significantly (*P* ≤ 0.05) longer (10.7 ± 0.1 cm) than those of Plainsman V (9.2 ± 0.4 cm), and consisted of 25.4 ± 1.7 and 17.9 ± 1.2 spikelets, respectively. No significant HD-stress dependent reduction in spike length was observed in either of the genotypes. Irrespective of the genotype and treatment, the grain number and fertility of florets developing in the upper part of the spikes were significantly lower than the relevant parameters of the basal florets. Although combined stress had no effect on the fertility ratio of Plainsman V, the fertility of HD-stressed Cappelle Desprez florets located in the base and top regions showed a significant decrease of 39% and 56%, respectively. Slight, but not significant HD stress-induced decreases and increases in TGW occurred in the basal and top florets, respectively, of both genotypes. HD stress only induced a non-significant 8% loss in plant production of Plainsman V. In contrast, as a consequence of the loss in fertility, the production of Cappelle Desprez was severely reduced by 55% (Table 2). According to the yield components recorded after co-stress during microgametogenesis, Plainsman V and Cappelle Desprez were considered as HD-tolerant and HD-sensitive varieties, respectively.

**Table 2:** Changes in yield components of the main spikes after HD stress in the winter wheat varieties Plainsman V and Cappelle Desprez.

Yield components	Spike half							
	
	Plainsman V				Cappelle Desprez			
		
	Control		HD stress		Control		HD stress	
				
	Base	Top	Base	Top	Base	Top	Base	Top
Spikelet no.	9.5 ± 0.4^b^	8.7 ± 1.0^b^	9.3 ± 0.3^b^	8.5 ± 0.6^b^	13.3 ± 1.3^a^	12.1 ± 0.7^a^	12.7 ± 0.7^a^	12.1 ± 0.9^a^
Grain no.	17.4 ± 1.8^c^	14.0 ± 2.6^de^	16.9 ± 1.0^cd^	14.2 ± 0.9^e^	27.4 ± 0.7^a^	20.7 ± 1.4^b^	15.4 ± 1.1^e^	8.9 ± 1.6^f^
Fertility (%)	61.4 ± 6.9^a^	53.8 ± 6.2^bc^	60.8 ± 2.0^ab^	50.6 ± 1.5^c^	66.8 ± 3.7^a^	55.8 ± 4.2^bc^	40.7 ± 0.5^d^	24.4 ± 2.6^e^
TGW (g)	42.3 ± 6.5^a^	38.0 ± 5.7^a^	37.7 ± 5.3^a^	39.0 ± 7.0^a^	40.1 ± 5.0^a^	31.3 ± 5.4^a^	27.3 ± 1.6^a^	33.5 ± 2.9^a^
Production (g)	0.73 ± 0.03^b^	0.52 ± 0.05^cd^	0.64 ± 0.12^b^	0.50 ± 0.08^d^	1.10 ± 0.15^a^	0.65 ± 0.13^bc^	0.42 ± 0.12^de^	0.30 ± 0.06^e^


*Values represent means ± standard deviations. In each row, means with different superscripts are significantly different at least at the *P* ≤ 0.05 level of probability. Base, lower half of the spike; HD, simultaneous heat and drought; Top, upper half of the spike; TGW, thousand-grain weight.*

### Floret Position, Truncation of Bracts, and HD-Triggered Stigma Dysfunction Influenced Fertility

In order to assess the effect of the stigma dysfunction triggered by HD stress on fertility loss in the sensitive genotype, a pollination experiment was conducted on Cappelle Desprez plants, where the effect of floret position, floret truncation and manipulation was also considered. Floret location (top or base of the spike) had a significant effect on fertility in both control and HD-stressed Cappelle Desprez plants (Table 3). The cutting off one-third of the bracts (glumes, paleas, lemmas) in the primary florets and the removal of the central florets (truncation, group iii) from the control spikelets caused a position-independent loss in fertility (base: 21%, top: 25%). Compared to the truncated and free-pollinated control florets (group iii), the hand pollination of truncated and emasculated (manipulated, group iv) control florets with control pollen did not reduce fertility. Compared to the free-pollinated intact control florets (group ii), HD stress induced a significant 62% and 33% loss in the fertility of truncated free-pollinated (group iii) and manipulated and hand-pollinated (group iv) florets, respectively (Table 3). The removal of one-third of the bracts had a similar fertility-reducing effect in HD-treated florets (base: 24%; top: 26%) as in the control. In free-pollinated HD-stressed spikes, there was a significant difference between the fertility loss in florets located in the base (45%) and top (56%). Both pistil- and-pollen dependent fertility loss varied significantly with position, being 22% and 36% in florets located in the upper halves of the spikes and 15% and 32% in basal florets, respectively (Table 3).

**Table 3:** Effect of floret position, floret manipulation, and hand pollination with control pollen on fertility rates of the Cappelle Desprez variety.

	Fertility % of the spikes with florets					
	
	Intact free pollinated (ii)		Truncated and free pollinated (iii)		Manipulated and hand pollinated (iv)	
			
	Base	Top	Base	Top	Base	Top
Control	75.6 ± 3.9^a^	61.6 ± 0.2^b^	59.9 ± 3.3^bc^	48.5 ± 0.3^d^	61.1 ± 1.0^b^	51.7 ± 0.2^c^
HD stress	41.4 ± 2.4^e^	27.4 ± 2.4^fg^	31.6 ± 0.3^f^	20.5 ± 0.1^g^	52.0 ± 0.5^c^	40.5 ± 2.7^e^


*Values represent means ± standard deviations. Means with different superscripts are significantly different at least at the *P* ≤ 0.05 level of probability. Base, lower half of the spike; HD, simultaneous heat and drought; top, upper half of the spike; truncated florets, the top third of the glumes, paleas and lemmas in all the primary and secondary florets were cut off and the central florets were removed 3 days before anthesis; manipulated florets, truncated and emasculated 3 days before anthesis; emasculation, removal of stamens.*

### Changes in Phenology Due to Combined Stress

The adverse environmental conditions significantly shortened the duration of microgametogenesis, which lasted for 7 days under optimum conditions. Independently of the genotype, HD-stressed plants started flowering 3 days earlier. Moreover, HD stress shortened the duration of grain filling in both Plainsman V and Cappelle Desprez, by 10 and 14 days, respectively.

### Effect of Co-stress on Spike and Anther Morphology and Pollen Viability

**FIGURE 2:**
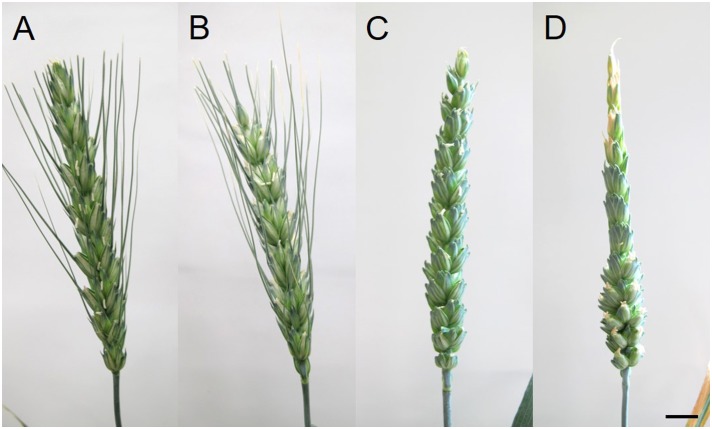
Spikes of **(A,C)** control and **(B,D)** HD-stressed **(A,B)** Plainsman V, and **(C,D)** Cappelle Desprez varieties 7 days after anthesis (DAA). **(D)** Note sterile florets located in the upper half of HD-stressed Cappelle Desprez spike. Bar represents 10 mm.

Compared to their respective controls (Figure 2A,C), there was no change in spike morphology in Plainsman V a week after HD treatment (Figure 2B), while the bracts of the apical florets in treated Cappelle Desprez spikes turned yellow and the spikes showed strongly reduced fertility (Figure 2D and Tables 2, 3). The size of the anthers did not vary significantly with the treatment.

**FIGURE 3:**
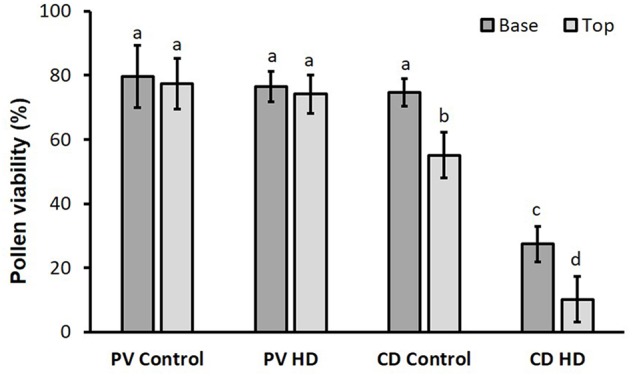
Viability of control and HD-stressed Plainsman V and Cappelle Desprez pollen in anthers located in the basal and top half of the spikes. Columns represent means; vertical bars denote ± standard deviations. Different letters above columns indicate significant difference between means at least at the *P* ≤ 0.05 level of probability.

The mean viability of Plainsman V pollen (80.4% ± 2.7) was independent of the treatment or floret position. In contrast, for the genotype Cappelle Desprez, lower pollen viability was observed in superior spikelets than inferiors under both optimum conditions and heat and drought co-stress. Compared to the base (74.6% ± 4.3) the viability of control pollen located in the top anthers was significantly (26%) lower. HD stress had a severe effect on Cappelle Desprez pollen; compared to their respective controls, viability of HD stressed pollen cells decreased by 63% and 81% in basal and apical anthers, respectively (Figure 3).

### Morphological and Anatomical Changes Induced by HD Stress in Pistils

**FIGURE 4:**
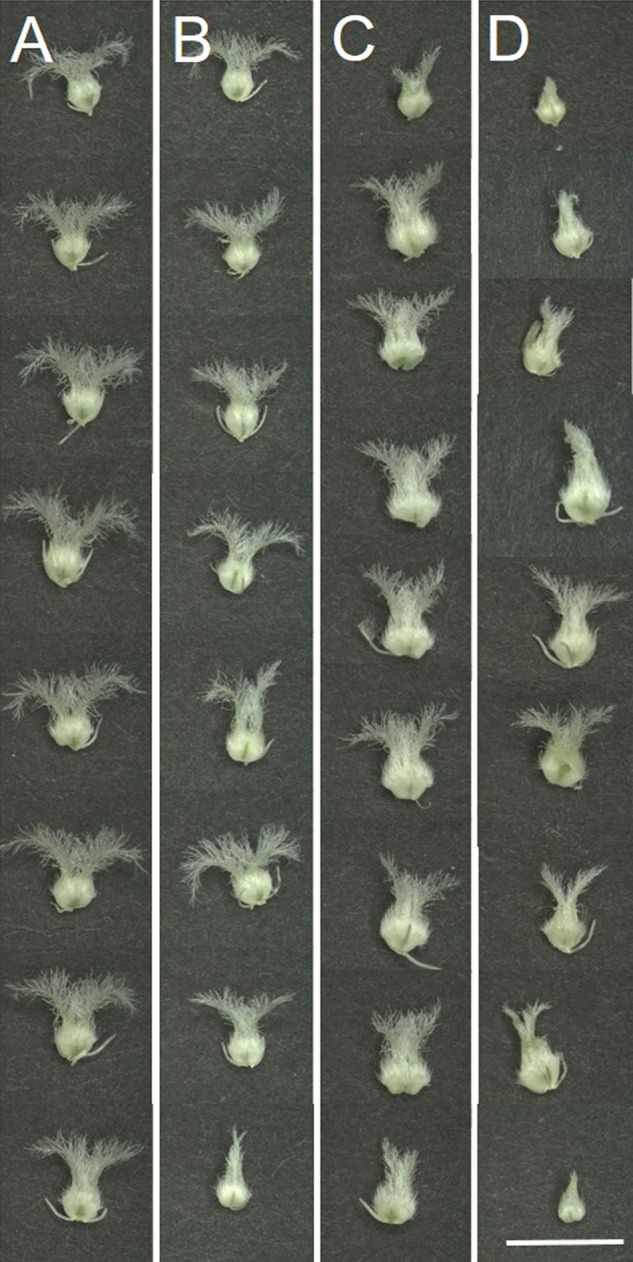
Morphology of female reproductive organs (pistils) dissected from **(A,C)** control and **(B,D)** HD-stressed **(A,B)** Plainsman V and **(C,D)** Cappelle Desprez florets at anthesis (representative images). Note that control stigmas are long with numerous secondary stigma branches, while HD-stressed thin stigmas are shorter with dehydrated, withered secondary branches typical of pistils located in the top of the spike. Bar represents 5 mm.

Although neither the morphology (Figure 4), nor the size (data not shown) of the ovaries changed with the treatment, compared to their respective controls (Figure 4A,C), the length of HD-stressed stylodia (Figure 4B,D) was significantly reduced in both genotypes (Table 4). The extent of genotype-independent reduction varied with floret position, averaging 35% at the base, 12% in the center and 31% at the top of the spikes. Some of the HD stressed Cappelle Desprez stylodia were shriveled, with fewer secondary branches (Figure 4).

**Table 4:** Stigma length in control and HD-stressed Plainsman V and Cappelle Desprez pistils at anthesis.

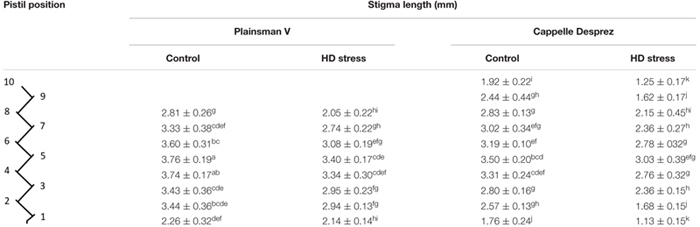

*Values represent means ± standard deviations. Means with different superscripts are significantly different at least at the *P* ≤ 0.05 level of probability. HD, simultaneous heat and drought.*

**FIGURE 5:**
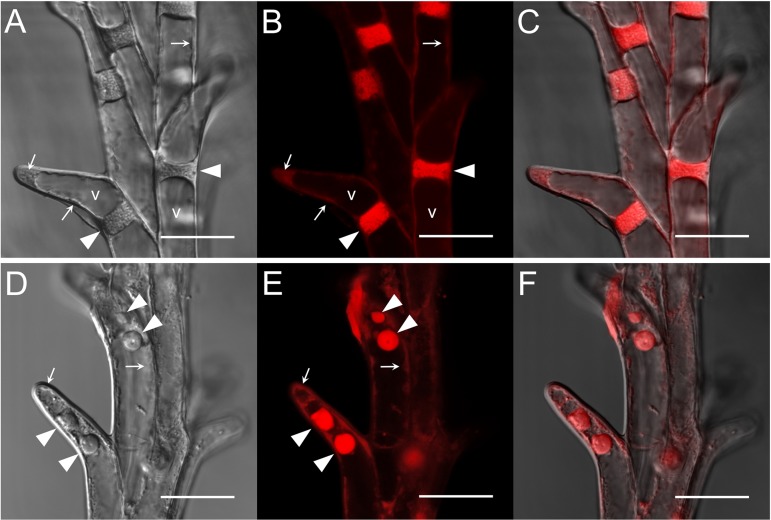
**(A–C)** Control and **(D–F)** HD-stressed stigmatic papilla cells of the stress-sensitive Cappelle Desprez variety at anthesis. Images were taken both in **(A,D)** differential interference contrast (DIC) and **(B,E)** fluorescent mode after staining with a SYTO-63 nucleic acid probe. Merged images are shown in **(C,F)**. Arrows, marginally located cytoplasm; arrowhead, nucleus; v, vacuole. Note fragmented nuclei in HD-stressed stigmatic papilla cells **(D–F)**. Bar represents 20 μm.

Compared to the control, no HD stress-induced structural alterations were observed in Plainsman V stigmatic papilla cells. In contrast, as a consequence of HD stress, the papilla cells of Cappelle Desprez partially lost their turgor and thus the secondary stigma branches were shriveled. Light microscopic studies using DIC and Syto 63 staining revealed the structural effects of HD treatment on the stigmatic papillae (Figure 5). Untreated stigmatic papilla cells possessed disk-shaped nuclei wedged tightly in between the large vacuoles that occupied almost the whole of the cell (Figure 5A–C). The cytoplasm was generally located on the periphery of the papilla cells, although large cytoplasmic segment was also found in control outwardly curved apical papilla cells. The stigmatic papilla cells of Cappelle Desprez pistils, especially those located in the top half of the spikes, showed signs of injury after treatment (Figure 5D–F). The nuclei were reshaped and relocated from their original position. Syto 63 staining revealed fragmentation of the nuclei and cytoplasm (Figure 5D–F).

**FIGURE 6:**
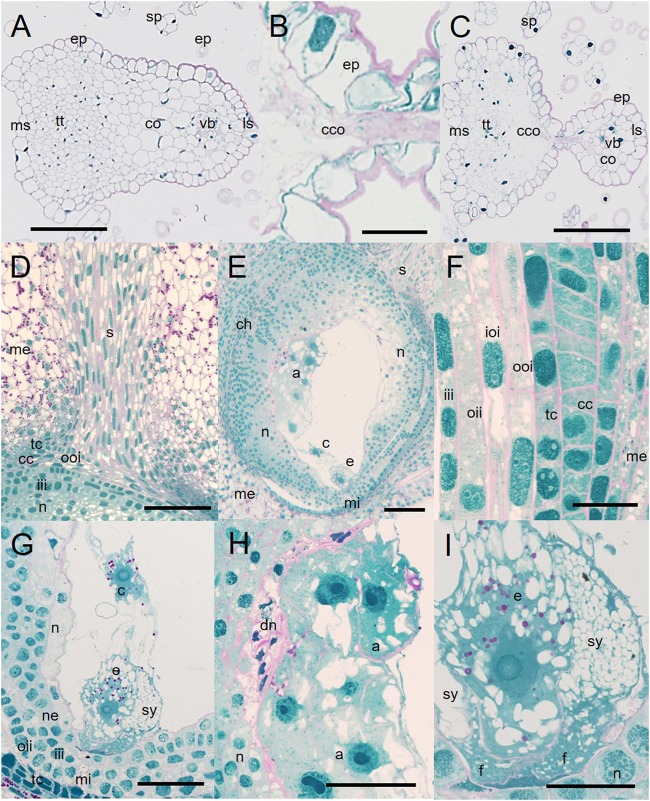
Micromorphology of non-fertilized Cappelle Desprez stylodia and ovules. **(A)** Cross-sectioned shield-like control stylodium halfway along the entire length. **(B)** Crushed mass of degenerated cortical cells in HD-stressed stylodia. **(C)** Structure of a HD-stressed bilobed stylodium. **(D)** Transmitting tissue surrounded by the mesocarp, with the ovule beneath it. **(E)** Egg cell, central cell, and antipodals located in a sagittally sectioned ovule lined with a position-dependent amount of nucellar cell layers. **(F)** Cell layers encompassing the ovule. **(G)** Egg cell apparatus located at the mycropilar end of the ovule, joined to the central cell with cytoplasmic bridges. **(H)** Degrading cell layers of the nucellus associated with antipodal cells. **(I)** Egg cell apparatus consisting of the egg cell and the synergids. a, antipodals; c, central cell; cc, cross cell; ch, chalaza; co, cortical cells; cco, crushed cortical cells; dn, degraded nucellar cells; e, egg cell; ep, epidermis; f, filiform apparatus; iii, inner layer of the inner integument; ioi, inner layer of the outer integument; ls, lateral side of the stylodium; me, mesocarp; mi, micropyle; ms, medial side of the stylodium; n, nucellus; ne, nucellar epidermis; oii, outer layer of the inner integument; ooi, outer layer of the outer integument; s, stylodium; sp, stigmatic papilla cells; sy, synergid; tc, tube cell; tt, transmitting tissue; vb, vascular bundle; pink coloration, carbohydrates; blue coloration, proteins. Scale bar represents **(A–D,H)** 100 μm; **(F,G)** 50 μm; **(I)** 20 μm.

The transversally cut surface of both control and HD-stressed stylodia was somewhat ovate at a quarter of the way from the top, and consisted of large, vacuolated cortical cells (co) surrounding a few small, cytoplasm-rich transmitting cells (tt). Although HD stress had no effect on the structure of Plainsman V stylodia, whether isolated from the top or base of the spike, those in the upper part of Cappelle Desprez spikes were slightly dehydrated. Halfway down the spike, control stylodia and those of HD-stressed Plainsman V had a shield-like shape (Figure 6A). A massive vascular bundle (vb) surrounded by cortical cells was located on the lateral side of the stylodia, the medial side consisted of small, turgid, well-demarcated transmitting cells with round nuclei. Multiple layers of large, vacuolated cortical cells connected the two sides of the stylodium (Figure 6A). By contrast, as a consequence of the HD stress-induced extensive degeneration of almost all the cortical cells and part of the transmitting tissue, a mass of crushed cells was visible in HD-stressed stylodia isolated from the top half of the sensitive genotype (Figure 6B), and as a consequence of the damage these turned into dumbbell-shaped bilobed structures (Figure 6C). A few intact cortical cells were visible exclusively in the proximity of the vascular bundle.

Irrespective of the genotype and treatment, unilocular ovaries and ovules showed similar anatomy in both control and HD-treated florets. No signs of dehydration were observed in these organs. The style (s), protruded into the ovary consisted of 10–15 rows of vacuolated spindle-like cells with large ovate nuclei. At the base the style took the form of a funnel that ended between the tube cell (tc) layer and the outer layer of the outer integument (oii; Figure 6D). The ovaries were well developed; the mesocarp cells (me) accumulated a large amount of starch (Figure 6D,E). The cross and tube cell layers were discontinuous at the base of the two styles, where the cells of the style were loosely connected to the outer layer of the outer integument (Figure 6D). Chloroplasts were visible in the dense cytoplasm of both the cross and tube cell layers (Figure 6F), but chloroplasts accumulating starch deposits were only visible in a few cell rows lining the base of the style. The ovary contained a single embryo sac surrounded by integuments (Figure 6E,F). The double layer of the outer integument was highly vacuolated (Figure 6F). The two layers of the inner integument formed the micropyle (mi) at the base of the ovule (Figure 6E,G). The ovules were lined with the nucellar epidermis (ne) and nucellus (n; Figure 6G). However, none of these were present in the vicinity of the micropyle, while large quantities of nucellar cells were observable at the chalaza (ch; Figure 6E). Irrespective of the treatment, degraded nucellar cells (dn) were found in the proximity of vacuolated antipodal cells (a; Figure 6H). The egg apparatus consisted of two highly vacuolated synergids (sy) with well-developed filiform apparatus (f), and the egg cell (e; Figure 6G,I). Regardless of whether the two polar nuclei of the central cell (c) were adjacent to the egg cell or not, they were attached to it by means of cytoplasmic bridges (Figure 6G). Both the egg cell and the central cell accumulated starch (Figure 6I).

### Influence of HD Stress on ROS and RNS Generation in the Stigma

**FIGURE 7:**
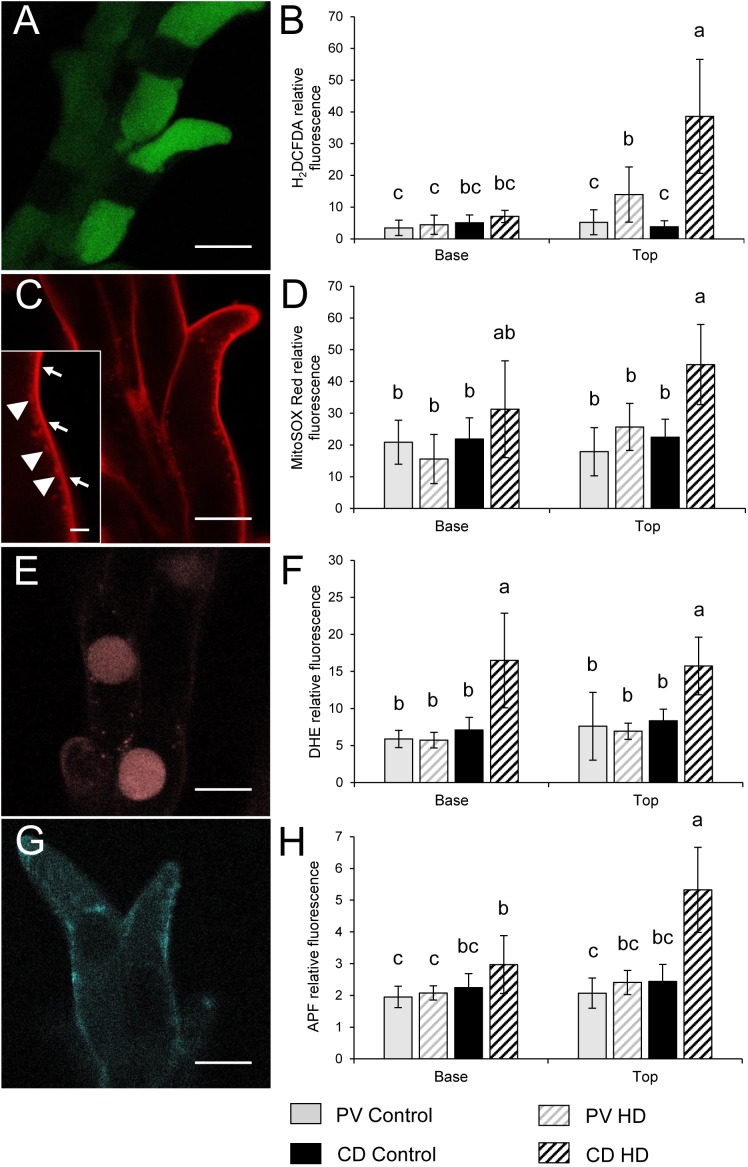
Localization of **(A,C,E,G)** fluorochromes and **(B,D,F,H)** relative fluorescence of stigmatic papillae cells observed after labeling with **(A,B)** H_2_DCFDA, **(C,D)** MitoSOX Red, **(E,F)** DHE and **(G,H)** APF in stigma papilla cells of control and HD-stressed Plainsman V and Cappelle Desprez wheat plants at anthesis. Specificity of used ROS probes: H_2_DCFDA, total ROS; MitoSOX Red, mitochondrial O_2_^-^; DHE, cytosolic O_2_^-^; APF, intracellular highly reactive OH and ONOO^-^. Arrows, specific MitoSox Red signal from the mitochondria; arrowheads, non-specific autofluorescence from the cell wall. In each histogram, letters above columns indicate significant differences between means at the *P* ≤ 0.05 level of probability. Bar represents **(A,C,E,G)** 10 μm and (**C** inset) 1 μm.

The ROS and RNS production of control and HD stressed stigmatic papilla cells was analyzed in both genotypes. In order to estimate the general oxidative stress manifested in the cells, non-fluorescent H_2_DCFDA, an indicator of total ROS was added to control and HD-stressed stigmas. H_2_DCFDA, after cleavage by oxidation was converted to the highly fluorescent 2′,7′-dichlorofluorescein. The fluorescence of the oxidized H_2_DCFDA showed vacuolar localization (Figure 7A). Irrespective of the genotype a weak fluorescent signal was observed in all the control stigmas and in those located in the lower half of HD-stressed spikes. In contrast, significantly higher fluorescence was detected in HD-stressed papilla cells dissected from the upper florets of both varieties: a more than two- and ten-fold significant (*P* ≤ 0.05) increase was observed in Plainsman V and Cappelle Desprez stigmas, respectively (Figure 7B).

Mitochondrial O_2_^•-^ production was assessed by MitoSOX Red staining. The specific signal of the oxidized fluorophore was detected in mitochondria located in the narrow, peripheral cytoplasm of the papilla cells (Figure 7C). There was no significant difference between the control and HD-treated stigmas in either spike halves of Plainsman V in terms of mitochondrial superoxide accumulation. In contrast, mitochondrial O_2_^•-^ generation was significantly increased in the upper stigmas of treated Cappelle Desprez (Figure 7D). Relative amount of the cytoplasmic superoxide radical was measured using DHE, which was detected in the nucleus after oxidation in the cytosol. Although the fluorescent signal was observed mainly in the nucleus, small cytoplasmic objects emitting superoxide-dependent fluorescence were also detected, presumably due to the incorporation of oxidized DHE into mitochondrial DNA (Figure 7E). Irrespective of the genotype and floret position (Figure 7F), the generation of cytoplasmic O_2_^•-^ was significantly increased as a consequence of HD stress.

The amount of highly ROS was estimated by APF labeling, which allows the detection of hydroxyl radical (OH^•^) and peroxynitrite (ONOO^-^). Relatively less radical-specific fluorescence was observed in the case of APF if compared to other probes used in this study (Figure 7G). In Plainsman V stigmas, no significant elevation of the fluorescent signal was measured after HD treatment. In contrast, a strong, significant rise in specific fluorescence was measured in the top halves of Cappelle Desprez spikes (Figure 7H).

**FIGURE 8:**
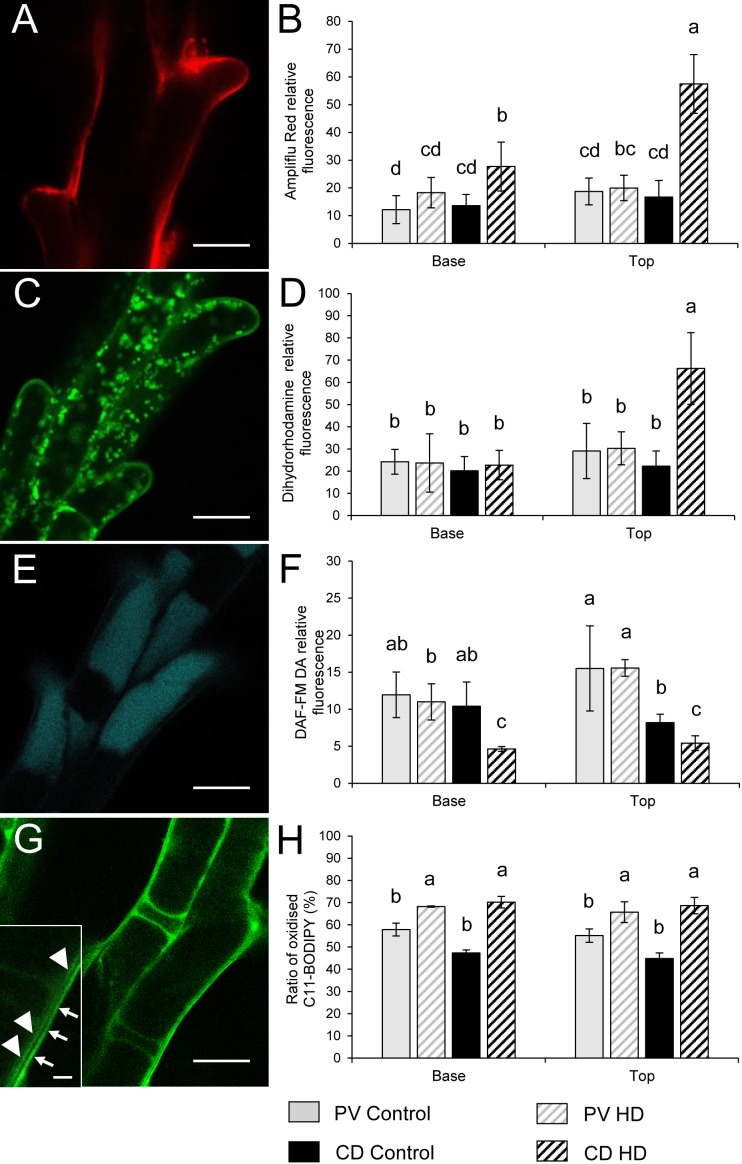
Localization of **(A,C,E,G)** ROS, RNS and peroxidized lipids and **(B,D,F,H)** relative fluorescence observed after Ampliflu Red **(A,B)**, DHR 123 **(C,D)**, DAF FM-DA **(E,F)** and C11-BODIPY^TM^ 581/591 **(G,H)** labeling in stigma papilla cells of control and HD-stressed Plainsman V and Cappelle Desprez wheat plants at anthesis. Ampliflu Red, DHR 123 and DAF FM-DA indicate the generation of extracellular H_2_O_2_, intracellular H_2_O_2,_ and nitric oxide, respectively. The intensity of C11-BODIPY^TM^ 581/591 labeling reveals the extent of lipid peroxidation. Arrows, specific C11-BODIPY^TM^ signal from the plasma membrane; arrowheads, non-specific autofluorescence from the cell wall. In each histogram, letters above columns indicate significant differences between means at the *P* ≤ 0.05 level of probability. Base, lower half of the spike; top, upper half of the spike. Bar represents **(A,C,E,G)** 10 μm and (**G** inset) 1 μm.

The relative content of extracellular hydrogen peroxide was measured using Ampliflu Red. A specific fluorescent signal of resorufin, the product of the reaction between H_2_O_2_ and Ampliflu Red was detected in the apoplast (Figure 8A). HD stress had no effect on the apoplastic hydrogen peroxide content of Plainsman V papilla cells, while co-stress induced a significant increase in apoplastic resorufin fluorescence at both the base and top of Cappelle Desprez spikes (Figure 8B). The intracellular generation of H_2_O_2_ and peroxynitrite was monitored with dihydrorhodamine 123, which after oxidation to rhodamine 123 (RH) accumulates in the mitochondrial membranes (Figure 8C), reflecting the H_2_O_2_ and peroxynitrite content of the cell. The RH fluorescence did not change significantly in Plainsman V papilla cells, while it showed a nearby threefold increase in stigmas located in the upper half of HD-treated Cappelle Desprez spikes (Figure 8D).

The relative content of nitric oxide (NO) was detected using a DAF-FMDA probe. NO was shown to be localized in the vacuoles of stigmatic papilla cells (Figure 8E). Compared to the control, HD stress had no effect on the nitric oxide accumulation in Plainsman V. However, a significant drop in the NO content of Cappelle Desprez papilla cells was observed, irrespective of the position of the florets in the spike (Figure 8F).

### HD Stress Induced the Peroxidation of Membrane Lipids

Lipid peroxidation was evaluated using a C11-BODIPY^TM^ probe, which showed a specific staining pattern localized in the membranes of papilla cells (Figure 8G). Treatment increased the oxidation of probes incorporated into the membrane in both genotypes, but to a different extent. On average, the proportion of oxidized C11-BODIPY^TM^ rose by 18% and 48% in Plainsman V and Cappelle Desprez, respectively (Figure 8H).

### Correlation Between Reactive Compounds and Fertility Loss

Very strong or strong negative correlations were found between the fertility ratio of the genotypes and the total ROS content (*r* = -0.85), extracellular H_2_O_2_ content (*r* = -0.94), intracellular H_2_O_2_ content (*r* = -0.84), OH^•^ and ONOO^-^ content (*r* = -0.91), mitochondrial O_2_^•-^ content (*r* = -0.91), cytoplasmic O_2_^•-^ content (*r* = -0.47) and lipid peroxidation (*r* = -0.60). A very strong positive correlation was found between the cytoplasmic O_2_^•-^ content and lipid peroxidation, while very strong correlations were detected between the generation of intracellular H_2_O_2_ (*r* = 0.98), mitochondrial O_2_^•-^ (*r* = 0.93), OH^•^ ONOO^-^ (*r* = 0.92) and extracellular H_2_O_2_. A moderate positive correlation (*r* = 0.55) was found between the fertility ratio and intracellular NO content of the papilla cells.

## Discussion

High temperature and drought often occur simultaneously during plant development causing severe yield loss in most wheat-growing areas. Structural and functional anomalies occurring as a consequence of environmental stress during reproductive processes have a serious influence on the success of fertilization and thus on yield production.

As in many members of the *Poaceae* family, the stigma of wheat is dry and plumose (Figure 1B) and the pistil is bifurcated. The primary branches, known as stylodia, are densely covered with multiseriate secondary branches consisting of papilla cells. While no signs of dehydration or anatomical anomalies were observed in the pistils of the tolerant genotype, moderately dehydrated secondary stigmatic branches were typical of HD-stressed Cappelle Desprez pistils isolated from the upper half of the spikes. A similar phenomenon was observed in wheat and sorghum stigmas when exposed to heat stress *per se* (Prasad and Djanaguiraman, 2014; Djanaguiraman et al., 2018). Moreover, the nuclei of stigmatic papilla cells changed their position and the nuclei and cytoplasm were fragmented. The stylodia of the sensitive genotype were malformed, as the majority of the cortical cells and some of the transmitting cells were crushed (Figure 6). No such environmental stress-induced structural anomalies have been described in angiosperms so far. A genotype-independent reduction in the stylodium length was detected in both HD-sensitive and -tolerant wheat genotypes after HD treatment, probably as a consequence of the arrested cell enlargement induced by a significant decrease in plant RWC. This contrasts with the findings of Jagadish et al. (2010) and Pan et al. (2018), who reported unaffected stigma length and stigma exsertion, respectively, after high temperature stress.

Heat and drought co-stress had no effect on the ovule or female gametophyte development in either of the wheat genotypes studied, in contrast to the findings of Saini et al. (1983), who reported that the embryo sacs were completely absent or formed abnormally with incomplete cellular organization and altered ovary development (reduced nucellus development, overproliferated integuments) in pistils subjected to continuous exposure to 30°C for 3 days during meiosis. The effect of water withdrawal on wheat ovule development is rather controversial. As found here, Saini and Aspinall (1982) reported that water withdrawal *per se* had no effect on ovule development. In contrast, Onyemaobi et al. (2017) considered that female reproductive organs could be one of the major contributors to low seed set in wheat stressed during meiosis. It is important to note that the male and female gametophytic processes in *Triticum aestivum* are not synchronized after meiosis and that the differentiation of the octonucleate embryo sac (female gametophyte) proceeds far more rapidly than that of its male counterpart (Tímár et al., 1997). It can be assumed that the 7-celled female gametophyte was already formed by the time the microspores entered the binucleate stage of development, which means that the development of the female gametophyte was accomplished by the second day of HD stress, which is why the treatment had no negative effect on the structure of the ovules.

No reduction in anther length was observed after HD stress. Nevertheless, in agreement with reports of the pollen viability-reducing effect of high temperature or drought (De Storme and Geelen, 2014), HD stress severely reduced pollen viability by 63% and 81%, in the basal and top halves, respectively, of treated spikes of the sensitive genotype. The competition for assimilates between the upper and basal spikelets is a well-known phenomenon that could be more exacerbated when the plants encounter drought stress (Chen et al., 2013), high temperature stress (Fu et al., 2016) and their combination.

The analysis of yield components revealed that Cappelle Desprez suffered significantly greater loss in plant production after treatment, so this variety was considered as sensitive to HD co-stress. The data indicate that the stress-induced damage to stigmatic papilla cells in Cappelle Desprez, which was verified by the results of anatomical observations in the present study, strongly contributed to the decrease in function and fertility. Spike fertility, which determines grain number and sink strength, is a crucial factor in wheat yield potential (Reynolds et al., 2009). Heat and drought stress significantly decrease fertility (Prasad et al., 2011), which is generally considered to be the consequence of the damage sustained by the male gametophyte, while the role of pistil dysfunction in yield loss is somewhat underestimated. The loss observed in the fertility of the HD-stressed sensitive genotype in this study highlighted the fact that not only the extensively studied meiotic processes, but also development of sexual organs taking place during gametogenesis is highly sensitive to a changing climate. Apart from the great sensitivity shown by pollen development to heat and drought stress (Saini and Aspinall, 1981; Saini et al., 1984; Lalonde et al., 1997; Jäger et al., 2008; for reviews, see Dolferus et al., 2011; De Storme and Geelen, 2014), the results of the pollination experiment demonstrated that the damage to female reproductive organs induced by HD stress reduced their functionality and was responsible for 34% of gross fertility loss. It might be expected that decreased fertility would be negatively correlated with TSW due to compensation. This was not true of Plainsman V, in which neither fertility nor TGW was influenced by the treatment. In contrast, the fertility and TGW of Cappelle Desprez dropped significantly both in the basal and top floret positions as a consequence of HD which indicates that this variety was unable to compensate for the reduced grain number by an increase in grain weight. Moreover, although the duration of grain fill was shortened by 10 and 14 days in the tolerant and sensitive genotype, respectively, the former gave a plant production similar to the control, while the production of the latter dropped in both spike halves. Prasad et al. (2011) reported a similar decrease in the number of days to physiological maturity of bread wheat cultivars under a combination of drought and high temperature stress applied at heading.

As oxidative stress is an important source of damage when plants face high temperature and water shortage simultaneously (Zandalinas et al., 2018), it can be assumed that ROS-mediated injury is a major factor contributing to structural changes. It was demonstrated here, that structural anomalies triggered by the HD-induced generation of ROS and RNS may stand in the behind of reduced stigma function and female-dependent fertility loss. In photosynthetic tissues, the main sources of ROS are the chloroplast and the peroxisome, especially when conditions are unfavorable (Waszczak et al., 2018). However, as photosynthesis does not occur in stigmatic papilla cells, major ROS-generating processes such as the reduction of O_2_ in photosystem I or photorespiration in the peroxisomes are absent. The potential ROS-generating regions in papilla cells are therefore the mitochondria, the glyoxysomes and the plasma membrane-cell wall-apoplast system (Noctor et al., 2014; Singh et al., 2016; Waszczak et al., 2018). Fluorescent ROS indicators provide powerful tools for the investigation of oxidative stress in living cells. Nevertheless, the majority of these probes have limitations due to their insufficient ROS specificity, which should be taken into account when interpreting the results (Ortega-Villasante et al., 2016). The present study confirmed that the stigmatic papillae of monocotyledonous wheat generate high amounts of ROS (O_2_^•-^, OH^-^, H_2_O_2_) and RNS (ONOO^-^, NO) at anthesis. These results are in accordance with the findings of McInnis et al. (2006), Serrano et al. (2010), and Zafra et al. (2016) who observed the accumulation of ROS/H_2_O_2_ in dicotyledonous angiosperms. The assessment of general cellular oxidative stress using H_2_DCFDA indicated a good correlation with fertility loss, implying that the high level of oxidants detected in the stigmatic papillae was located in the top half of Cappelle Desprez spikes. The amount of oxidants in Plainsman V rose to a smaller extent, which did not lead to fertility loss.

The ROS metabolism in plant cells is an intricate network involving many enzymatic and metabolite elements (Noctor et al., 2015). The first type of ROS generated by the electron transport chain in mitochondria is superoxide (Rhoads et al., 2006). Unfortunately, the monitoring of superoxide with DHE and MitoSOX Red is impaired by the two-electron oxidation of these dyes by other oxidants (Wang et al., 2013), making these probes unsuitable for the selective detection of O_2_^•-^. Nevertheless, the similar sensitivity and the organelle selectivity of these probes make them ideal for the quantitative comparison of the oxidants present in the mitochondria and cytoplasm (Zielonka and Kalyanaraman, 2010). The control level of these oxidants was fourfold higher in the mitochondria compared to the cytoplasm, confirming the leading role of mitochondria in ROS generation. HD stress triggered a sharp rise in mitochondrial oxidant generation exclusively in Cappelle Desprez, while the cytoplasmic amount of these compounds rose significantly in both genotypes, irrespective of floret position. This suggests that although Plainsman V possesses a more efficient mitochondrial electron transport chain and/or ROS scavenging system than Cappelle Desprez, there are other cytoplasmic oxidant-generating mechanisms which produce similar levels of oxidizing agents in the cytoplasm of both varieties. A very strong positive correlation was found between the cytoplasmic O_2_^•-^ content and lipid peroxidation.

Superoxide is converted by superoxide dismutase enzyme (SOD) into hydrogen peroxide, a ROS with a long lifespan (Waszczak et al., 2018). H_2_O_2_ is able to diffuse through membranes either alone (Lim et al., 2016) or facilitated by aquaporins (Bienert and Chaumont, 2014), and may thus reach other parts of the cells. In the present study DHR 123 was used to monitor intracellular hydrogen peroxide, but as it also reacts with peroxynitrite (Crow, 1997), the results must be handled with care. DHR 123 diffuses freely into the cells, where it is oxidized to Rhodamine 123, which selectively stains mitochondria (Johnson et al., 1980). Hence, although the fluorescent signal is detected in the mitochondria, it represents the H_2_O_2_ and peroxynitrite concentration of the whole cell. The results showed that the cytoplasmic concentration of these compounds was only elevated in the top half of Cappelle Desprez spikes. In non-photosynthesizing cells, the hydrogen peroxide level may be elevated not only by mitochondrial activity, but also through the glyoxylate cycle in glyoxysomes. This pathway allows the turnover of membrane lipids as well as the synthesis of various hormones playing important roles in the stress response, like indole acetic acid, jasmonic acid and salicylic acid (Corpas et al., 2015). Higher expression of acyl-CoA oxidase, an enzyme that generates H_2_O_2_ in the glyoxylate cycle, was reported in drought-treated Arabidopsis plants (Bray, 2002). It can be hypothesized that the glyoxylate cycle became more active in the upper florets of Cappelle Desprez, contributing to the elevated H_2_O_2_ level in papilla cells. Increased amounts of apoplastic H_2_O_2_, determined with Ampliflu Red, were observed exclusively in treated Cappelle Desprez papilla cells. Apoplastic ROS is generated actively by enzymatic mechanisms, such as apoplastic polyamine oxidases and respiratory burst oxidase homologs localized in the plasma membrane (reviewed by Waszczak et al., 2018). According to the literature, apoplastic hydrogen peroxide is involved in drought and salt stress acclimation (Miller et al., 2010), although a more important role is proposed in intercellular signal transduction (Choudhury et al., 2017). The higher apoplastic levels of H_2_O_2_ in Cappelle Desprez may be explained by the occurrence of more severe water shortage in this genotype, which may induce elevated amounts of signaling molecules. Very strong correlations were found between the generation of intracellular H_2_O_2_, mitochondrial O_2_^•-^, OH^•^, ONOO^-^, and apoplastic H_2_O_2_.

The amounts of highly reactive oxidative and nitrosative compounds, hydroxyl radical (OH^•^) and peroxynitrite (ONOO^-^), respectively, were estimated using APF (Setsukinai et al., 2003). The results showed that the amount of these compounds elevated only in Cappelle Desprez papillae following HD stress, especially in those located in the top half of the spikes. In living cells, hydroxyl radical (OH^•^) is formed from H_2_O_2_ through the Fenton reaction catalyzed by iron and other transition metals (Rhoads et al., 2006). Despite its short lifetime, OH^•^ has a significant role in lipid peroxidation (Pham-Huy et al., 2008; Noctor et al., 2015). Peroxynitrite (ONOO^-^), another highly reactive radical which can oxidize APF, is generated by the reaction of O_2_^•-^ with NO (Arasimowicz-Jelonek and Floryszak-Wieczorek, 2011). This short-lived RNS takes part in the oxidation and nitration of various molecules, such as DNA, lipids, and proteins (Vandelle and Delledonne, 2011). The amount of hydrogen peroxide, a precursor molecule of OH^•^, closely mirrored the presence of the APF signal in the present experiment, which was not true for the precursors of peroxynitrite, NO and O_2_^•-^. These data imply that the APF signal is potentially more indicative of hydroxyl radicals than peroxynitrite in this experiment.

The nitric oxide content and fertility of Cappelle Desprez were found to be lower in the top than in the basal halves of control spikes. After HD treatment, both values dropped in this genotype, irrespective of floret position, while Plainsman V showed no significant change. An increasing number of scientific papers report the significance of NO in fertilization. Seligman et al. (2008) demonstrated the NO-generating activity of stigmatic tissue in Arabidopsis. Moreover, an Arabidopsis mutant proven to be defective in NO production showed reduced fertility, which was restorable using a NO donor compound (Guo et al., 2003). The present results confirm the proposed link between the HD stress-triggered drop in nitric oxide content and reduced fertility in monocotyledonous plants. This link may be the role that nitric oxide plays in pollen tube guidance. Prado et al. (2008) showed that NO acts as a negative chemotropic agent of pollen tube growth, providing a tool for maternal tissues to route pollen tubes toward the ovule. Positive correlation between fertility and NO content of stigmatic papilla cells indicates that this compound promotes successful fertilization in wheat.

Oxidative damage during abiotic stress originates mainly from the alteration of lipids and proteins by oxidative compounds in the cell (Anjum et al., 2015). The ratio of oxidized to reduced Cll-BODIPY 581/591, a fluorescent membrane lipid homolog, increased to a higher extent in Cappelle Desprez than in Plainsman V after HD treatment. Moreover, a very strong positive correlation was found between the cytoplasmic O_2_^•-^ content and lipid peroxidation. It should be noted that the oxidation of Cll-BODIPY 581/591 can only be initiated by a variety of oxy-, peroxy-, or hydroxyl radicals, but not by superoxide, nitric oxide, or hydroperoxides (Pap et al., 2000), so the fluorescent signal intensity of the probe is not adequately proportional to lipid peroxidation. However, this fluorophore can be used to estimate the general oxidation of membrane lipids.

It can be assumed that the hostile microenvironment induced by the generation of ROS and ONOO^-^ and the decrease in NO production prevented successful pollen–pistil interactions, resulting in fertilization failure in a significant number of Cappelle Desprez florets. Although there seems to be a close correlation between high levels of fluorescence from ROS-sensitive probes and fertility loss, the exact cause and mechanisms of ROS-derived injury and the drop in nitric oxide content are still not clear due to the low specificity of the fluorescent probes currently available.

## Conclusion

This is the first report on the effect of HD co-stress on female reproductive cells and organs in wheat. Overall, this research revealed that HD co-stress reduced the RWC of wheat plants and increased ROS and ONOO^-^ generation, decreased NO production and enhanced lipid peroxidation in stigmatic papilla cells. These changes induced alterations in the morphology and anatomy of female reproductive organs and shortened the duration of gametogenesis and grain filling, with the combined effect of significantly reduced fertility and plant production to an extent dependent on floret position and tolerance. Further investigations will be needed to shed light on the genetic background of the differences observed between the studied genotypes. A better understanding of the effect of HD stress and the mechanisms of tolerance may lead to the development of wheat genotypes suitable for future climatic conditions.

## Data Availability

All datasets generated for this study are included in the manuscript and/or the Supplementary Files.

## Author Contributions

KJ conceived and designed the experiments. AF, ES, GS-E, BB, and KJ, performed the experiments. AF and KJ carried out statistical analysis and wrote the manuscript. All authors read and reviewed drafts of the manuscript and approved the final manuscript.

## Conflict of Interest Statement

The authors declare that the research was conducted in the absence of any commercial or financial relationships that could be construed as a potential conflict of interest.

## Footnotes

1 http://www.wheatpedigree.net/sort/show/49608

2 http://www.wheatpedigree.net/sort/show/10022
