# Supplementary material for: Stigma Functionality and Fertility Are Reduced by Heat and Drought Co-stress in Wheat
Source: Front Plant Sci. 2019 Mar 7;10:244. doi: 10.3389/fpls.2019.00244 (PMC6417369; doi:10.3389/fpls.2019.00244)
Supplement: Supplementary file 1 [file Table_1.docx]

Supplementary Material

**Stigma Receptivity and Fertility are Reduced by Heat and Drought Co-stress in Wheat**

Attila Fábián, Eszter Sáfrán, Gabriella Szabó-Eitel, Beáta Barnabás and Katalin Jäger*

*** Correspondence:** Katalin Jäger: jager.katalin@agrar.mta.hu

Supplementary Table 1: Negative and positive control treatments used during the quantification of ROS, RNS content and lipid peroxidation

| Fluorescent probe | Application | Negative control (scavenger) | Negative control  (enzyme inhibitor) | Positive control |
| --- | --- | --- | --- | --- |
| H_2_DCFDA | General cellular oxidative stress redox marker | 200 U/ml CAT + 1 mM ASA, 2 mM TMP | 10 uM DPI, 10 mM Na-azid | Microwave 10” |
| MitoSOX Red | Detection of mitochondrial superoxide | 2 mM TMP | 10 um DPI, 10 mM Na-azid | Microwave 10” |
| DHE | Detection of intracellular superoxide | 2 mM TMP | 10 um DPI, 10 mM Na-azid | Microwave 10” |
| APF | Detection of highly reactive ROS, RNS | 1,25 uM D-mannitol | - | Microwave 10” |
| Ampliflu Red | Detection of extracellular H_2_O_2_ | 200 U/ml CAT + 1 mM ASA | 10 uM DPI, 10 mM Na-azid | 10 mM H_2_O_2_ |
| DHR 123 | Detection of intracellular H_2_O_2_ | 200 U/ml CAT + 1 mM ASA | 10 uM DPI, 10 mM Na-azid | 10 mM H_2_O_2_ |
| DAF-FMDA | Detection of intracellular nitric oxide | 100 uM cPTIO | 5 mM L-name | 100 uM SNAP |
| BODIPY ^581^⁄_591_C11 | Estimation of lipid peroxidation | 2 mM TMP, 200 U/ml CAT + 1 mM ASA | 10 uM DPI, 10 mM Na-azid | Microwave 10” |

Abbreviations, suppliers:

ASA: ascorbic acid, SIGMA

CAT: catalase, SIGMA

cPTIO: (4-Carboxyphenyl)-4,4,5,5-tetramethylimidazoline-1-oxyl-3-oxide, SIGMA

DPI: Diphenyleneiodonium chloride, SIGMA

L-NAME: Nω-Nitro-L-arginine methyl ester hydrochloride, SIGMA

TMP: 2,3,5,6-Tetramethylpyrazine, SIGMA
